# Glucose uptake via GLUT1 maintains T cell survival during proliferative stress

**DOI:** 10.1186/1753-6561-6-S3-P33

**Published:** 2012-06-01

**Authors:** Andrew N Macintyre, Valerie A Gerriets, E Dale Able, Jeffery C Rathmell

**Affiliations:** 1 Department of Pharmacology and Cancer Biology, Department of Immunology and Sarah W. Stedman Center for Nutrition and Metabolism, Duke University Medical Center, Durham, NC 27710, USA; 2 Division of Endocrinology, Metabolism and Diabetes and Program in Molecular Medicine, University of Utah School of Medicine, Salt Lake City, UT84132, USA

## Background

Lymphocyte survival is regulated via the balance between pro- and anti-apoptotic BH3 family proteins. *In vitro* this balance is highly dependent on glucose availability. *In vivo*, T-lymphocytes develop in the thymus and then exit to the periphery, where they continually migrate until they encounter cells presenting viral/bacterial antigens. This encounter activates the T cell and induces both proliferation and differentiation into functionally mature T cell subsets. The role of glucose metabolism in regulating cell survival during each of these stages of the T cell life cycle is still unclear; however, we have recently demonstrated that different T cell functional subsets demonstrate distinct metabolic profiles. *In vivo* manipulation of T cell glucose metabolism may therefore represent a novel strategy to manipulate immune responses. In order to explore this area we generated mice with a T cell specific deletion of GLUT1, a major glucose transporter in T cells.

## Materials and methods

Mice containing loxP flanked *Slc2a1* (encoding GLUT1) [1] were crossed with mice expressing *Cre* under a T cell specific *Lck* promoter. Mice with tandem *myc* tags knocked into an exofacial loop of GLUT1 were generated in-house. Biochemical, metabolic profiling and *in vivo* proliferation assays were performed as described [2].

## Results

GLUT1 expression was assayed during T cell development, T cell activation and in mature T cells. Expression of GLUT1 was limited to lymphocytes undergoing rapid proliferation, with only developing and activated T cells exhibiting surface expression of GLUT1. Across the mature T cell subsets, immunosuppressive regulatory T cells (Treg) demonstrated far lower GLUT1 expression in comparison to pro-inflammatory effector T cells (Teff). These differences correlated with differing rates of glucose consumption. Deletion of GLUT1 from developing T cells using a *LckCreSlc2a1^fl/fl^* mouse model caused a severe reduction in the number of T cells in both the thymus and periphery. One exception to this was the Treg cells, the relative proportion of which increased. Naïve T cells lacking GLUT1 were viable, however when induced to proliferate either *in vitro* or *in vivo* they were unable to correctly upregulate their glucose metabolism, resulting in a misbalance of BH3 family proteins and induction of cell death.

## Conclusions

Tight regulation of the glucose transporter GLUT1 is required for normal T cell development and activation. GLUT1 mediated glucose transport is required to drive T cell proliferation and to maintain cell survival. One exception to this is the Tregs, which are far less dependent on GLUT1 for survival. Manipulating GLUT1 mediated glucose metabolism may therefore represent a novel therapeutic strategy to skew T cell responses *in vivo.*
